# Sympathetic, Metabolic Adaptations, and Oxidative Stress in Autism Spectrum Disorders: How Far From Physiology?

**DOI:** 10.3389/fphys.2018.00261

**Published:** 2018-03-22

**Authors:** Antonietta Messina, Vincenzo Monda, Francesco Sessa, Anna Valenzano, Monica Salerno, Ilaria Bitetti, Francesco Precenzano, Rosa Marotta, Francesco Lavano, Serena M. Lavano, Margherita Salerno, Agata Maltese, Michele Roccella, Lucia Parisi, Roberta I. Ferrentino, Gabriele Tripi, Beatrice Gallai, Giuseppe Cibelli, Marcellino Monda, Giovanni Messina, Marco Carotenuto

**Affiliations:** ^1^Department of Experimental Medicine, Section of Human Physiology and Unit of Dietetics and Sports Medicine, Università degli Studi della Campania “Luigi Vanvitelli”, Naples, Italy; ^2^Department of Clinical and Experimental Medicine, University of Foggia, Foggia, Italy; ^3^Clinic of Child and Adolescent Neuropsychiatry, Department of Mental Health, Physical and Preventive Medicine, Università degli Studi della Campania “Luigi Vanvitelli”, Naples, Italy; ^4^Department of Health Sciences, University “Magna Graecia”, Catanzaro, Italy; ^5^Child Neuropsychiatry, Department of Psychology and Pedagogical Sciences, University of Palermo, Palermo, Italy; ^6^Childhood Psychiatric Service for Neurodevelopmentals Disorders, Chinon, France; ^7^Department of Surgical and Biomedical Sciences, University of Perugia, Perugia, Italy

**Keywords:** autism spectrum disorders (ASD), Orexin-A, oxidative stress, heart rate (HR), heart rate variability (HRV)

## Abstract

Autism spectrum disorders (ASD) is a complex and multifaceted neurobehavioral syndrome with no specific cause still identified, despite the worldwide increasing (prevalence for 1,000 children from 6.7 to 14.6, between 2000 and 2012). Many biological and instrumental markers have been suggested as potential predictive factors for the precocious diagnosis during infancy and/or pediatric age. Many studies reported structural and functional abnormalities in the autonomic system in subjects with ASD. Sleep problems in ASD are a prominent feature, having an impact on the social interaction of the patient. Considering the role of orexins (A and B) in wake-sleep circadian rhythm, we could speculate that ASD subjects may present a dysregulation in orexinergic neurotransmission. Conversely, oxidative stress is implicated in the pathophysiology of many neurological disorders. Nonetheless, little is known about the linkage between oxidative stress and the occurrence or the progress of autism and autonomic functioning; some markers, such as heart rate (HR), heart rate variability (HRV), body temperature, and galvanic skin response (GSR), may be altered in the patient with this so complex disorder. In the present paper, we analyzed an autism case report, focusing on the rule of the sympathetic activity with the aim to suggest that it may be considered an important tool in ASD evaluation. The results of this case confirm our hypothesis even if further studies needed.

## Background

Autism spectrum disorders (ASD) is a complex and multifaceted neurobehavioral syndrome with no specific cause still identified, despite the worldwide increasing (prevalence for 1,000 children from 6.7 to 14.6, between 2000 and 2012). Many biological and instrumental markers have been suggested as potential predictive factors for the precocious diagnosis during infancy and/or pediatric age. Functional magnetic resonance imaging (fMRI) has shown the structural abnormalities in relevant brain structures such as the amygdala, cingulated anterior cortex, and cerebellum (Uddin and Menon, 2009). These alterations seem to be associated with neurotransmitters dysregulation with the imbalance between excitation and inhibition in neural circuits (Purcell et al., 2001a,b).

Many studies reported structural and functional abnormalities in the autonomic system in subjects with ASD (Neri et al., 2009a,b; Bujnakova et al., 2016; Bonaventure et al., 2017). As previously described, ASD patients show decreased levels of essential fatty acids. In this scenario, the assumption of polyunsaturated fatty acids (PUFA) can help the brain development and function (Brigandi et al., 2015).

On the other hand, ASD patients present sleep disorders, such as parasomnias, obstructive apnea sleep disorders syndrome (OSAS), disorders of initiating and maintaining of sleep, sleep-related movement disorders (Giallongo et al., 2011; Accetta et al., 2016; Precenzano et al., 2017). Considering the role of orexins (A and B) in wake-sleep circadian rhythm, we could speculate that ASD subjects may present a dysregulation in orexinergic neurotransmission also involved in various brain dysfunctions connected with numerous neuropsychiatric disorders including neurodevelopmental disorders. Sleep disturbance in ASD patients depending on the increased activity of the orexinergic system (probably due to amygdala dysfunction) associated with a reduction of 5-HT and melatonergic system activity (Kohyama, 2016; Mondola et al., 2016; Petito et al., 2016; Bertozzi et al., 2017). Orexin evaluation may be considered a new interesting biomarker in ASD pathogenesis, even if to date only associative studies were performed to demonstrate their relationship with health disturbances, such as obesity (Hao et al., 2017).

Conversely, oxidative stress is implicated in the pathophysiology of many neurological disorders (Essick and Sam, 2010). The diacron reactive oxygen metabolites (d-ROMs) test has been used in the clinics, to evaluate the oxidative stress. Particularly it could be considered an important atherosclerotic risk factor in type 2 diabetes mellitus (T2DM), in cardiac disease, neurological disorders, aging, and cancer (Kotani et al., 2013). Nonetheless, little is known about the linkage between oxidative stress and the occurrence or the progress of autism and autonomic functioning; some markers, such as heart rate (HR), heart rate variability (HRV), body temperature, and galvanic skin response (GSR), may be altered in patient with this so complex disorder (Bricout et al., 2017; Hufnagel et al., 2017; Kuiper et al., 2017; Oshodi et al., 2017).

HRV is considered, indeed, a standard noninvasive method for evaluating Autonomic Nervous System (ANS) function (Messina et al., 2012; Neri et al., 2013). It was frequently investigated in a large number of cardiology studies. Nowadays, recent scientific studies suggested the HRV as an important tool in the field of physiology, psychology, psychiatry, and occupational medicine. The HRV variations are linked with the sympathetic and parasympathetic activities directed to the sinus node characterized by each cardiac cycle. The high frequency (HF) component is associated solely with parasympathetic activity, while the low frequency (LF) component is associated with both sympathetic and parasympathetic activities, even if the sympathetic activity is the greater contributor.

In the present paper, we analyzed an autism case report, focusing on the rule of the sympathetic activity with the aim to suggest that it may be considered an important tool in ASD evaluation.

## Case presentation

We enrolled a male, a child 9 years old, height 1.33 m and weight 28 kg (normal BMI), in the Child Neuropsychiatry at the University of Palermo. The values of serum thyroid hormones levels, systolic and diastolic blood pressure have remained within the normal range.

Written informed consent was obtained from parents. At any time, they could choose to leave the study. All the investigation procedures were approved by the local Human Ethical Review Committee in accordance with the revised Declaration of Helsinki (2013).

The pregnancy was normally conducted and completed with a natural birth at 38 gestational weeks. The infant conditions were good. The development stages were achieved in time (walking at 11 months; first words at 12 months, two phrase sentences at 15 months). Between 15 and 18 months, there was no evolution of language. During this period, sleeping difficulties (rarely sleepwalking) and bizarre behaviors (hand flapping, turn on himself) were reported. At the age of about 3 years old, parents noticed the tendency to isolation, lack of expressive language, difficult eye contact, stereotyped movements of the body, hyperactivity.

At the age of 4 years the “Social Interaction Disorder” was diagnosed. The neuropsychomotor therapy (play-therapy) and speech therapy for 2 times per week were prescribed. These therapies helped the patient to improve his clinical situation.

At the age of 6 years, the complete diagnosis was done: “Cognitive disability in the subject with psychopathological aspects of autism spectrum and signs of immaturity.” In this scenario, the FRAXA (Fragile X) test was performed, considering that it is a very important test for anyone with signs of autism or unexplained developmental delay. The genetic FRAXA test was negative.

Clinical evaluation, instrument examinations, plasma Orexin-A detection, and d-ROMs test were performed. All data were in accord with the diagnosis of autism in a subject with intellectual disability.

### Clinical evaluation

The patient had poor language skills: he communicated using one or two sentences of words even if he sang in full sentences (echolalics). The comprehension was good as he was able to hyperventilate for 3 min. Frequently, he related to others mostly by pointing. He showed self-stimulating behaviors in the form of rocking, manual rotation, and hand flapping. His motor skills were below normal. His sleep was good but he was defiant and unable to calm down at bedtime. He did not show any interest in other children and his eye contact is poor. He did not show aggressive behavior, but often he did afinalistic actions (turn around the room).

For the diagnosis, the ADOS-2 (Autism Diagnostic Observation Schedules, Second Edition) was performed. The total score of the “Module 1” was compatible with autistic spectrum, with a total score of 22 (cut-off for autistic spectrum = 8; cut-off for autism = 12). Comparison Scoring ADOS-2 also indicated that the children presented a high level of autism spectrum-related symptoms compared to children with ASDs of the same age and language level.

Furthermore, the semi-structured interview ADI-R was carried out. As summarized in Table 1, all results were compatible with ASD.

**Table 1:** The results of semi-structured interview ADI-R.

	**Patient score**	**Cut-off for autism**
Qualitative abnormalities in mutual social interaction	15	10
Qualitative communication abnormalities	14	7
Constrained, repetitive and stereotyped behavior patterns	10	3

Finally, the (CarS-T) Infant Caring Assessment Scale was carried out: the patient had a total score of 45, that was indicative of a relation compromisation and it was compatible with “Severely Autistic.”

## Laboratory investigations and diagnostic tests

### Electroencephalographic evaluation (EEG)

The cerebral background rhythm during awake stage showed well-organized and well-developed average voltage 9–10 Hz alpha activity predominantly in the posterior regions. With eye-opening, it was bilaterally synchronous and symmetrical. No spike-and-wave discharges or any lateralizing abnormalities were observed. Photic stimulation did not produce any abnormalities. A brief drowsiness was seen in the later part of the recording. Hyperventilation was also performed for about 3 min; no abnormalities were seen during the procedure. In conclusion, the EEG test was normal and no epileptiform discharges or any other paroxysmal activities or focal abnormalities were observed.

### Magnetic resonance imaging (MRI) of the brain

The magnetic resonance imaging (MRIs) of the brain was performed: no any alteration of the brain was detected.

### HR measurement

The HR measurement was carried out with a chest strap wired to a digital R-R recorder (BTL08 SD ECG); the QRS-signal wave-form R-R signal was sampled at the resolution of 1 ms. The HR (beats min^−1^) was calculated using the formula: HR = 60/R-R interval in seconds; the R-R interval was converted into seconds.

In the patient, the HR-value was higher (84 bpm) than the normal value of children (average normal value 70 bpm).

### Power spectral analysis (PSA) of HRV

The Power spectral analysis (PSA) of HRV was evaluated by an electrocardiogram (ECG) for 5 min. The signals were acquired on a PC at 100 s/s by an electrocardiograph (delta-1 plus, Cardioline, Milan, Italy) connected to the serial port of a PC; a custom software made with LabView (National Instruments, Texas, USA) was used for data acquisition and analysis.

The R waves were automatically recognized, and the RR intervals were calculated and resampled to obtain a constant-time-based signal (100 ms). The Fourier transform was then applied to this signal and visualized in the form of power LF (0.04–0.15 Hz) and HF (0.15–0.40 Hz). The LF, HF, and the LF/HF ratio were used to estimate the sympathetic and parasympathetic activities. Although the time window for HRV recording is generally >5 min, the Task Force on HRV (1996) indicates that main spectral components are distinguished in a spectrum calculated from short-term recordings of 2–5 min.

In the patient, the LF (S^2^/Hz) was higher (0.74 S^2^/Hz) than the normal values (a.n.v. 0.44 S^2^/Hz).

### Galvanic skin response (GRS)

The GSR parameters were measured simultaneously using the SenseWear Pro Armband™ (version 3.0, BodyMedia, Inc. PA, USA), which was worn on the right arm over the triceps muscle at the midpoint between acromion and olecranon processes, as recommended by the manufacturer.

The GSR (μS) value in the patient was higher (1.79 μS) than the normal status (a.n.v. 1.11 μS).

### Rectal temperature

Rectal temperature was measured with electronic thermometer thermistor/thermocouple (Ellab A/S, Hilleroed, Denmark).

The body temperature changed in relationship with normal status. In the patient, the rectal temperature (°C) was higher (37.81°C) than the normal value (a.n.v. 37.01°C).

### Plasma Orexin-A detection

Blood sample was obtained at 8:00 a.m. after overnight fast into Vacutainer tubes (BD, Franklin Lakes, NJ) containing EDTA and 0.45 TIU/ml of aprotinin.

Each sample was mixed and then immediately centrifuged at 3,000 rpm for 12 min at 4°C.

Plasma was separated and stored at −80°C until analysis.

The plasma Orexin-A levels were measured with Hypocretin Orexin-A1 ELISA (Enzyme-Linked Immuno Assay) kit (Phoenix Pharmaceuticals). For this test, the minimal detectable concentration was 0.37 ng/ml, the intra-assay error <5% and the inter-assay error <14%.

Before ELISA test, the Orexin-A extraction was obtained with Sep-Pak C18 columns (Waters, Milford, MA) using the following protocol:

- 10 ml of methanol and 20 ml of H_2_O were used to activate the columns;
- 1.5 ml of sample was added to the column and washing with 20 ml of water;
- the sample was eluted slowly with 80% acetonitrile and resulting volume was reduced to 400 μl under nitrogen flow;
- the aliquot obtained was led to exsiccation using Speedvac (Savant Instruments, Holbrook, NY).

To perform the ELISA test, the dry residue was dissolved in water.

There was no cross-reactivity of the antibody for hypocretin-1 (16–33), hypocretin-2, agouti-related protein (83–132)-amide.

The Orexin A-values (3,592 pg/mL) was higher in the patient than to the normal status (a.n.v. 2,100 pg/mL).

### d-ROMs (reactive oxygen metabolites) test

The d-ROMs test is a simple assay marketed for analyzing the total amount of hydroperoxides in serum via the Fenton's reaction.

Hydroperoxides, consisting of lipids, carbonylated proteins, and oxidized nucleic acids, are one of the most important ROS involved in oxidative stress and their measurement is considered a reliable marker of oxidation in plasma.

The test was performed with the Free Radical Analytical System 4 (FRAS 4): this is a photometric analytical system developed for the assessment of oxidative stress that measures plasma hydroperoxides concentrations using the d-ROMs test with a single drop of peripheral (finger) blood.

The blood sample was collected by patient's finger (0.15 mL) in a heparinized microcuvette. Thanks to the centrifuge of the FRAS 4 Evolvo System, plasma was immediately isolated by centrifugation at 37°C for 60 s. The plasma was dissolved in an acidic buffer (pH 4.8) in which its hydroperoxides react with the transition metal ions liberated from the proteins in the acidic medium and was converted to alkoxy and peroxy radicals. Subsequently, a colorless chromogen was added (N,N-diethyl-para-phenylenediamine). These newly formed radicals oxidized this chromogen that changed into a radical cation producing a magenta colored derivative. This color is directly correlated with the concentration of hydroperoxides in the plasma sample that is proportional to the quantity of ROMs, according to the Lambert-Beer law. The photometer FRAS 4 Evolvo (absorption at 505 nm, Temperature 37°C) was used to measure the magenta color in order to measure the hydroperoxide concentration. The d-ROMs value was expressed in the arbitrary unit U. CARR (Units Carratelli), as established by the manufacturer (1 U. CARR corresponds to 0.08 mg of H_2_O_2_/dL). Normal values range between 250 and 300 U. CARR and values higher than 300 U. CARR suggest increased oxidative stress (Cornelli et al., 2001).

The results of the d-ROMs test showed different values: in the patient the value (532 U) was higher than to the normal status (a.n.v. 255 U); this finding suggests the increase of oxidative stress in ASD.

## Discussion

The present paper may suggest the need to broaden horizons and the study target on ASD, including oxidative stress, neurotransmitters evaluation, and sympathetic activity measurements. As summarized in Table 2, the parameters tested in the case study are adulterated observing the normal values.

**Table 2:** Results of the parameters analyzed in the case study.

**Parameters**	**Values**
HR measurement	84 bpm (average normal value, a.v.n., 70 bpm)
Power spectral analysis (PSA) of HRV (LF)	0.74 S^2^/Hz (a.n.v. 0.44 S^2^/Hz)
Galvanic skin response (GRS)	1.79 μS (a.n.v. 1.11 μS)
Rectal temperature	37.81°C (a.n.v. 37.01°C)
Plasma Orexin-A detection	3,592 pg/mL (a.n.v. 2,100 pg/mL)
d-ROMs (reactive oxygen metabolites) test	532U (a.n.v. 255 U)

The oxidative stress is implicated in the pathophysiology of many neurological disorders, such as anxiety, depression, schizophrenia, bipolar disorder (Salim, 2014).

Undoubtedly the ASD pathogenesis is complex and still not well-identified, hypothesizing that autism can be traced back to a single and univocal pathogenesis. On the other hand, there is increasing evidence that ASD patients show excessive ROS production as reported by many studies (Ghezzo et al., 2013; Bafunno et al., 2014; Anwar et al., 2016; El-Ansary et al., 2017; Howsmon et al., 2017; Khemakhem et al., 2017; Oshodi et al., 2017).

In this perspective, the PUFA administration may be justified in ASD children (Bramanti et al., 2016; Parletta et al., 2016; Sun et al., 2016; Weiser et al., 2016).

Moreover, also vitamin D and its metabolites have been recognized as lower in ASD children respect of typically developing children (Basheer et al., 2017; Berridge, 2017; Jia et al., 2018), pinpointing the role of neuroinflammation in ASD etiology (Mostafa and Al-Ayadhi, 2012; Macfabe, 2013; Salomone et al., 2014; Cianci et al., 2016).

Neuroinflammation may explain the dysregulation between GABA and Glutamate cortical neurocircuitry in ASD children, particularly in the frontostriatal system with functional network topology (Cerame et al., 2008; Jakab et al., 2013; Carvalho Pereira et al., 2017; Naaijen et al., 2017; Nardone et al., 2017).

Conversely, these neurotransmitters alterations could explain also the sleep disorders such as nocturnal awakenings, insomnia, and parasomnias (Bramanti et al., 2012; Precenzano et al., 2017) and food selectivity in ASD children (Chistol et al., 2017; Li et al., 2017; Suarez, 2017). In this context may be explained the cerebral metabolism increasing (Mitelman et al., 2017) and the autonomic hyperfunctioning in ASD (Avola et al., 2004; Goodman, 2016; Parisi et al., 2017) sustained by high Orexin A levels (Messina et al., 2013, 2014, 2015; Kohyama, 2016; Messina A. et al., 2016; Messina G. et al., 2016).

Even if further studies needed, the findings of this study confirm the hypothesis that the markers of the sympathetic activity could become an important tool in ASD evaluation.

## Author contributions

AnM, VM, IB, FP, MaS, AgM, MR, LP, and RF: substantial contributions to the conception or design of the work, or the acquisition, analysis, or interpretation of data for the work. AV, MoS, RM, FL, FS, and SL: drafting the work or revising it critically for important intellectual content. FS, GT, BG, GC, and MM: final approval of the version to be published. AgM, GM, and MC: agreement to be accountable for all aspects of the work in ensuring that questions related to the accuracy or integrity of any part of the work are appropriately investigated and resolved. All authors read and approved the final manuscript.

### Conflict of interest statement

The authors declare that the research was conducted in the absence of any commercial or financial relationships that could be construed as a potential conflict of interest.
